# Research progress in the detection of trace heavy metal ions in food samples

**DOI:** 10.3389/fchem.2024.1423666

**Published:** 2024-05-28

**Authors:** Linxing Si, Qian Wu, Yulong Jin, Zhuo Wang

**Affiliations:** State Key Laboratory of Chemical Resource Engineering, College of Chemistry, Beijing Advanced Innovation Center for Soft Matter Science and Engineering, Beijing University of Chemical Technology, Beijing, China

**Keywords:** heavy metal ions, detection, food samples, nanomaterials, sensors

## Abstract

Food safety is the basis for ensuring human survival and development. The threat of heavy metal ions to food safety has become a social concern with the rapid growth of the economy and the accompanying environmental pollution. Some heavy metal ions are highly toxic even at trace levels and pose significant health risks to humans. Therefore, ultrasensitive detection of heavy metal ions in food samples is important. In this mini-review, recent advances in the analytical methods based on nanomaterials for detecting trace heavy metal ions in food samples are summarized in three categories: electrochemical, colorimetric, and fluorescent methods. We present the features and sensing mechanisms of these three methods, along with typical examples to illustrate their application in the detection of heavy metal ions in foods. This mini-review ends with a discussion of current challenges and future prospects of these approaches for sensing heavy metal ions. The review will help readers understand the principles of these methods, thereby promoting the development of new analytical methods for the detection of heavy metal ions in food samples.

## 1 Introduction

Food is the basis for human beings to maintain nutritional balance, carry out growth and development, and maintain physical health. Over the past decades, the development of the modern economy, wastewater irrigation, improper disposal of industrial wastes, and abuse of agrochemicals have led to the massive accumulation of heavy metal ions in agricultural soil and surface water (Ai et al., 2018; Mukherjee et al., 2021; Lai et al., 2023). Heavy metal ions have a long half-life, cannot be naturally degraded, and are enriched in living organisms (Keyster et al., 2020). Heavy metal ions in soil and surface water can easily accumulate in agricultural products through the biological chain. Heavy metal ions can reach a high level in the human body by eating and drinking, endangering human health (Zhang et al., 2017; Meng et al., 2023). Hence, heavy metal ions have become one of the most serious environmental and ecological pollutants.

Heavy metals are broadly defined as metallic elements with a relatively high density (>5 g/cm^3^). Toxic heavy metal elements mainly refer to mercury (Hg), cadmium (Cd), lead (Pb), chromium (Cr), arsenic (As), zinc (Zn), and et al. (Bansod et al., 2017). Among them, As is a non-metallic element, but it is listed as a toxic heavy element based on its chemical properties and toxicity. In general, even at relatively low concentrations, heavy metal ions can interact with nitrogen, sulfur, and oxygen of the biomolecules such as proteins and DNA. The interactions lead to disruption of the structure and function of these biomolecules, and ultimately inducing irreversible chronic or acute intoxication of the organisms (Shen et al., 2022). For example, Cd(II) disrupts iron homeostasis by inducing hyperactivation of heme oxygenase-1 and harassing lipid metabolism, ultimately leading to iron death (Zeng et al., 2021). Even at ultra-low daily doses, Cd(II) may gradually bioaccumulate along the food chain. The terminal half-life of Cd(II) in human organs is about 10–30 years (Guo et al., 2023). High levels of Cd(II) can cause kidney and liver damage and even induce cancer (Qin et al., 2021). Another highly toxic heavy metal ion is Pb(II), which damages almost all organs in the body (Hemmaphan and Bordeerat, 2022). The guideline value of the World Health Organization for the concentration of Pb(II) in drinking water is 4.83 × 10^−8^ M. Even if the concentration of Pb(II) in human blood is less than 5 μg/dL, it may trigger mental decline and behavioral difficulties in children. In short, heavy metal ions enter the human body along the food chain by contaminating soil, food, and water, ultimately leading to neurological disorders, digestive disorders, liver and kidney damage, immune system dysfunction, as well as cancer. Monitoring technology is the basis for preventing and combating heavy metal pollution. The quantity of heavy metal ions in foods is generally in the trace range. The maximum limit for heavy metal contamination in foods has been reported to be 0.02 mg/kg (Rachmawati et al., 2024). Therefore, the development of sensitive and efficient analytical methods for the determination of trace heavy metal ions in food samples is important for human health.

A wide variety of techniques have been applied to detect heavy metal ions in foods (Yang et al., 2023). The analytical methods have undergone a process of development from traditional analytical methods to instrumental analytical methods. Conventional analytical methods include inductively coupled plasma-mass spectrometry (ICP-MS) (Shih et al., 2019), atomic absorption spectrometry (AAS) (Kartoğlu et al., 2024) and gas chromatography-mass spectrometry (GC-MS) (Nozari et al., 2023). Many of these techniques offer excellent selectivity and detection limits and are capable of simultaneously measuring multiple heavy metal ions (Ding et al., 2021). However, these traditional analytical methods are complex to operate, requiring expensive and large analytical instruments, as well as experienced researchers (Yu et al., 2021). These methods are not suitable for rapid screening and on-site detection of trace heavy metal ions in foods. To solve these problems, many new analytical methods have been reported, such as electrochemical methods (Pan et al., 2024), colorimetric methods (Sharifi et al., 2022), and fluorescence methods (Chen et al., 2022). Compared with traditional analytical methods, these methods have the advantages of simple operation, high sensitivity, and high accuracy (Lim et al., 2021). Therefore, in the following sections, this mini-review highlights recent advances in these three analytical techniques (electrochemical, colorimetric, and fluorescent methods) for the detection of trace heavy metal ions in food samples from 2021 to 2024. In the end of the review, current challenges and future prospects of these approaches for sensing applications are briefly discussed.

## 2 Electrochemical sensing

**FIGURE 1:**
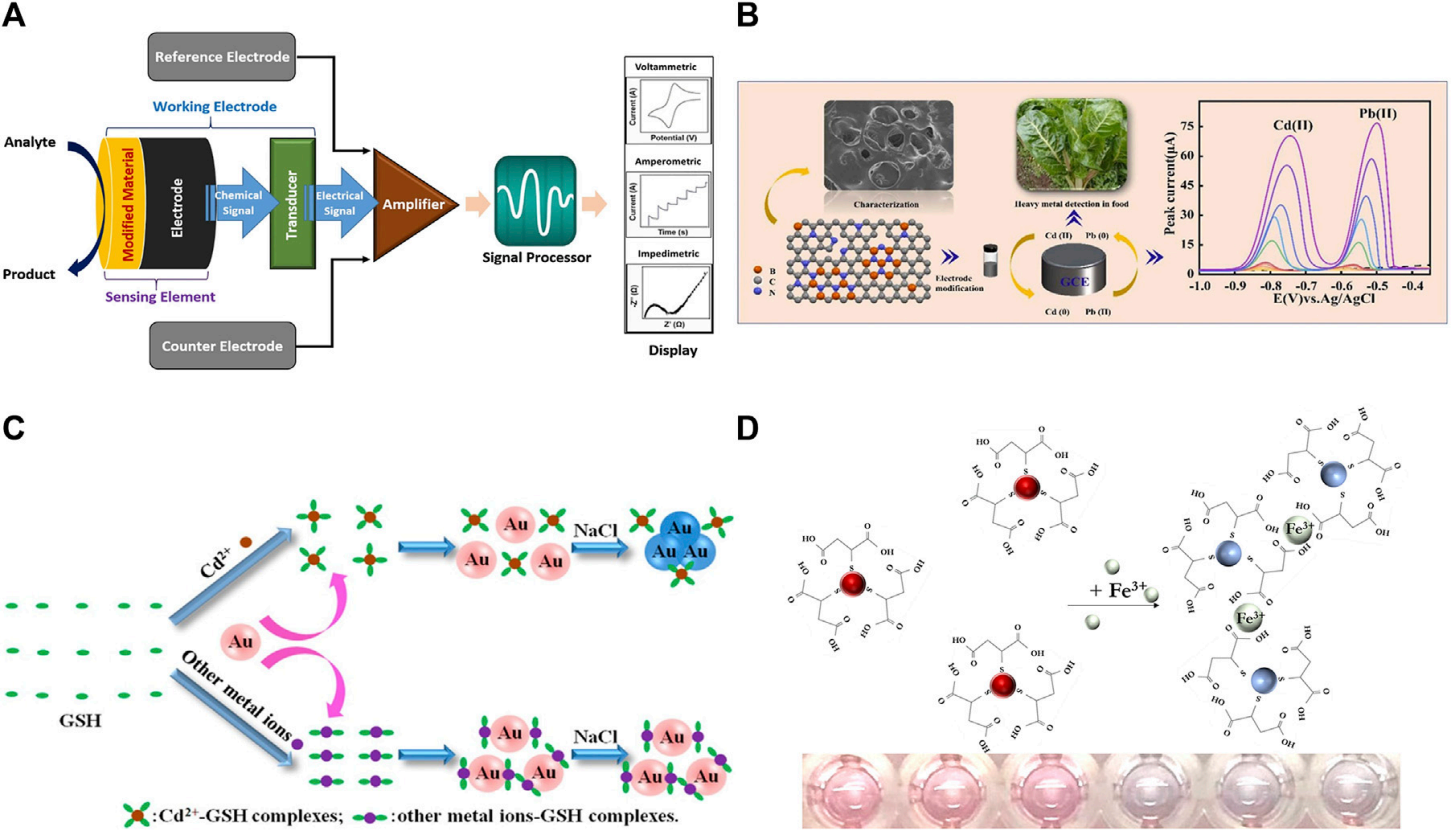
**(A)** Schematic diagram of an electrochemical sensor, reproduced with permission from (Amali et al., 2021). **(B)** Schematic diagram of BCN-Nafion/GCE detection of Cd(II) and Pb(II) in food samples, reproduced with permission from (Huang et al., 2023). **(C)** Schematic representation of the colorimetric detection of heavy metal ions using AuNPs, reproduced with permission from (Guo et al., 2014). **(D)** Scheme of colorimetric detection of Fe(III) ions using MSA-AuNPs, reproduced with permission from (Komova et al., 2021).

Electrochemical methods are analytical methods based on the electrochemical properties of measured substances in solution and at electrodes. An electrochemical sensing system usually consists of a counter electrode, a reference electrode, and a working electrode (Sardesai et al., 2020). The system indirectly detects heavy metal ions by measuring physical quantities such as electrode potential, charge, current, and conductivity. The principle of the electrochemical sensor is shown in Figure 1A (Amali et al., 2021). In chemical composition analysis, electrochemical analysis is a fast, sensitive, accurate, simple, and low-cost method for micro and trace analysis. Important progress has been made in the development of new electrode modification materials for the electrochemical detection of trace heavy metal ions, among which a series of complexes, such as MOF and MXene, have been widely used (Li et al., 2021).

Metal-organic frameworks (MOFs) are an extremely versatile type of super porous nanomaterials. With excellent chemical properties, rich and tunable microporous structure, and large specific surface area, MOFs showed unique advantages in the adsorption of heavy metal ions (Wang et al., 2022; Zhang et al., 2022). Researchers (Ma et al., 2024) synthesized a novel thiacalix [4]arene-based metal-organic framework Co-LTPA. Co-LTPA/GCE had a strong binding affinity for heavy metal ions and was used for the detection of Cd(II) and Pb(II) in foods (milk, honey, and orange juice), with a limit of detection (LOD) of 0.119 and 0.279 nM, respectively. MOFs were complexed with inorganic materials (such as carbon-based materials, metals, and metal oxides) to further improve the performance of electrode materials. For example, Dong et al. (2023) developed a sensing platform by integrating the material of MWCNTs and CeMOF for the simultaneous determination of Cd(II) and Pb(II) in grain and water samples, with the LOD of 2.2 and 0.64 ppb, respectively. Similarly, Huo et al. (2022) developed an electrochemical sensing platform that could simultaneously detect Cd(II), Pb(II), Cu(II), and Hg(II). The electrode was modified with a metal-organic backbone (UiO-66-NH_2_) and a carbon-based material, three-dimensional graphene (3DGO). UiO-66-NH_2_ had a large specific surface area and porous structure. The abundant amino groups on the surface of UiO-66-NH_2_ could bind with Cd(II), Pb(II), Cu(II), and Hg(II). 3DGO could increase the electron transfer rate and further improve the electrochemical properties of the composites. The 3DGO/UiO-66-NH_2_ electrochemical sensor was capable of simultaneously quantifying these heavy metal ions in food samples (rice, milk, and honey samples). Pang et al. (2023) modified amino-functionalized Co-based metal organoskeletons (Co-MOF-NH_2_) on electrodes deposited with gold nanoparticles (AuNPs) for the detection of Pb(II) and Cd(II). The developed Co-MOF-NH_2_/AuNPs/CPE could be used to detect real food samples (drinking water, juice, tea, grain, fruits, vegetables, liver and aquatic products), with the LOD of 7.0 × 10^−2^ and 1.1 × 10^−2^ ng mL^−1^ for Pb(II) and Cd(II), respectively. In order to further improve the reliability of the sensors and minimize the influence from the complex environment, ratiometric electrochemical sensors were developed (Yang et al., 2018). Wang et al. (2024) realized the proportional detection of Pb(II) and Hg(II) using differential pulse voltammetry signals of carbon dots (CDs) and UiO-66-CNTs as response and reference signals. The sensor could be applied for the detection of Pb(II) and Hg(II) in edible vegetables.

Zeolitic imidazolate frameworks (ZIFs) are a special class of MOF materials. Surface functionalization and heteroatom doping of ZIFs are important ways to improve their physicochemical properties. As illustrated in Figure 1B, Huang et al. (2023) prepared a boron and nitrogen co-doped porous carbon material (BCN) to improve the physicochemical properties of ZIF-8. The BCN-Nafion/GCE could be used for the detection of trace Cd(II) and Pb(II) in Beta vulgaris var. cicla L samples. Moreover, two-dimensional antimonene could be synthesized on the surface of ZIFs to improve the stability and biocompatibility of electrode materials. Zhang et al. (2023) prepared an electrochemical sensor based on ZIF-67@AMNFs by loading antimonene sheet on the surface of ZIF-67 MOF. ZIF-67@AMNFs electrochemical sensor was used for the simultaneous determination of Cd(II), Pb(II), and Hg(II) in milk, honey, and tea samples with the LOD of 0.01, 0.042, and 0.031 pM, respectively.

Novel transition metal carbides/carbonitrides (MXenes) have attracted researchers in the field of electrochemistry. Among all the discovered MXenes, titanium carbide (Ti_3_C_2_T_x_) aroused great attention (Qiu et al., 2022). Chen et al. (2023a) prepared amino-functionalized multilayer titanium carbide (NH_2_-Ti_3_C_2_T_x_). NH_2_-Ti_3_C_2_T_x_ was used to detect Cd(II) and Pb(II) in food samples including rice, wheat, sorghum and corn, with the LOD of 0.41 and 0.31 μg L^−1^, respectively. Researchers modified the composition and structure of MXene material to further enhance the electrode properties (Yu et al., 2019). A new strategy was applied to create highly interconnected porous structures, assembling 2D MXene nanosheets into 3D structures. Chen et al. (2023b) combined reduced graphene oxide (rGO) with melamine-doped MXene to obtain 3D composite aerogels with good electrochemical properties. The 3D melamine/MXene/rGO composite aerogel modified SPCE electrode could simultaneously detect Zn(II), Cd(II), and Pb(II) in foods (sorghum, rice, wheat and corn), with the LOD of 0.48, 0.45, and 0.29 μg L^−1^, respectively. Wen et al. (2022) constructed a MXA-CuO/CC electrochemical sensor for the simultaneous detection of Cd(II) and Pb(II) by combining MXene aerogels and CuO on the surface of flexible carbon cloth. In addition, this work utilized the synergistic adsorption of oxygen vacancies and Bi (III) to enhance the performance of the sensor. MXA-CuO/CC showed high reliability and efficiency for the detection of Cd(II) and Pb(II) in grain. Dong et al. (2024) coupled UiO-66-NH_2_ and MXene-rGO aerogel to prepare a novel sensing electrode for the simultaneous detection of ultra-trace Cd(II) and Pb(II) in grain and water samples.

## 3 Colorimetric sensing

The naked-eye detectable color change is an attractive feature of colorimetric sensors, and colorimetry can be detected quickly and conveniently (Nazifi et al., 2021). Heavy metal ions undergo a series of reactions with the substances, resulting in a change in the color of the solution. Qualitative and quantitative detection of heavy metal ions can be achieved by visual analysis or using an ultraviolet-visible spectrophotometer. Since each colorimetric material has its unique optical and chemical properties, they have completely different sensing mechanisms. In recent years, colorimetric sensors based on metal nanomaterials and nanoenzymes have been widely used for the detection of heavy metal ions.

The metal nanoparticles as colorimetric signaling probes has been applied on the basis of their localized surface plasmon resonance (Sönnichsen et al., 2005). The reasons that localized surface plasmon resonance sensor can cause color changes for two reasons. On the one hand, heavy metal ions can induce aggregation or dispersion of nanoparticles; on the other hand, heavy metal ions can induce morphological changes of nanoparticles. The basic principle is shown in Figure 1C (Guo et al., 2014). AuNPs are easily functionalized, visualized and coordinable than other metal nanoparticles, AuNPs have been extensively investigated for the detection of heavy metal ions with colorimetric signals. Hua et al. (2023) developed two convenient, instrument-free new strategies for the instantaneous determination of Cu(II) and Hg(II). The analytes promoted the reduction of chloroauric acid to different forms of AuNPs, resulting in different color variations. The nanosensors could be applied to detect Cu(II) and Hg(II) in drinking water with the LOD of 3.5 and 0.1 nM, respectively. Bhattacharyya and Hossain (2024) synthesized LGC-AuNPs using Lannea Grandis Coromandelica bark extract as reducing and stabilizing agent. The LGC-AuNPs-based sensor could produce color changes within 20 s for speedy and sensitive response to Zn(II) and Hg(II). LGC-AuNPs probe was used for the accurate detection of Zn(II) and Hg(II) in drinking water with extremely low detection limits. Some organic molecules were commonly used for the functionalization of metal nanoparticles to recognize heavy metal ions. As illustrated in Figure 1D, researchers (Komova et al., 2021) developed a simple method to prepare mercaptosuccinic-acid-functionalized gold nanoparticles (MSA-AuNPs). When Fe(III) was present, the functional groups (MSA) on the surface of AuNPs bind with these metal ions to form a large number of cross-linked structures, resulting in the aggregation of AuNPs, along with the color of the solution changing from red to blue-gray. MSA-AuNPs can be used to determine Fe(III) in drinking water, with a detection limit lower than the maximum allowable concentration for drinking water defined by the World Health Organization.

Recently, rapid detection of heavy metal ions based on nanozymes showed great potential. Nanozymes are a class of nanomaterials with biocatalytic function, which has the advantages of stable performance and are easy to be mass-produced (Jiang et al., 2019; Li et al., 2019). Hu et al. (2024) explored a colorimetric method based on a phosphatase mimic (MPA-CeO_2_) to analyze toxic Hg(II). When Hg(II) was present, the catalytically hydrolysis of colorless p-nitrophenyl phosphate to yellow p-nitrophenol by MPA-CeO_2_ was blocked. The method achieved the detection of Hg(II) in milk samples with the LOD as low as 0.16 nM. However, structural diversity and uneven elemental distribution of nanozymes resulted in low activity of nanozymes. To solve the problem, novel single atom nanozymes (SAzymes) have been continuously developed (Cheng et al., 2019). Song et al. (2022) demonstrated the oxidase-like activity of the prepared SAzyme (SACe-N-C nanozyme). Fe(III) and Cr(VI) significantly expedited the electron transfer rate of Ce(III) and Ce(IV) on the surface of the SACe-N-C nanozyme. Therefore, Fe(III) and Cr(VI) accelerated the SACe-N-C nanoenzymes-catalyzed process of substrate 3,3′,5,5′–tetramethylbenzidine. The sensor enabled rapid detection of Fe(III) and Cr(VI) in drinking water and spinach samples with the LOD of 34.72 and 93.65 ng mL^−1^, respectively.

## 4 Fluorescence sensing

The interaction between fluorescent probes and heavy metal ions can change the fluorescence characteristics of the probes, thus achieving the purpose of metal detection. Compared with traditional techniques, the fluorescence spectroscopic methods are characterized with fast response time, simple operation, high selectivity and sensitivity. Fluorescence spectroscopy is more suitable for the analysis of trace metal ions in complex matrices (Lian et al., 2020). For fluorescence analysis, the choice of fluorescent probes is a key factor. A wide variety of fluorescent probes have been applied. In this section, we briefly discuss advances in fluorescent nanomaterials for the detection of heavy metal ions in food samples.

Fluorescent nanomaterials with unique photoluminescent properties have gained considerable interest. The fluorescent probes can be categorized into: semiconductor quantum dots (QDs), carbon nanomaterials, and metal nanoparticles. Semiconductor QDs including CdS, CdSe, CdTe, CdS and ZnSe were widely used in the fields of metal ion detection (Anas et al., 2019). He et al. (2022) prepared an “on-off” sensor based on the glutathione (GSH)-modified CdS QDs for the detection of Cu(II), Hg(II), and Mg(II) in foods. The fluorescence of GSH-CdS QDs sensor was linearly quenched by Cu(II), Hg(II), and Mg(II). The LOD were 0.22, 5.8, and 1.6 ng mL^−1^, respectively. Hu et al. (2023) used GSH-CdTe QDs for Cu(II) detection, where the concentration of Cu(II) showed a good linear relationship with the fluorescence attenuation of the sensor in the range of 20–1100 nM. GSH-CdTe QDs had been successfully applied to the detection of real samples such as hawthorn, American ginseng and honeysuckle, allowing detection of Cu(II) with the LOD of 10.12 nM. However, the cytotoxicity and hydrophobicity of semiconductor QDs still face significant challenges as ideal fluorescent probes in food samples.

**FIGURE 2:**
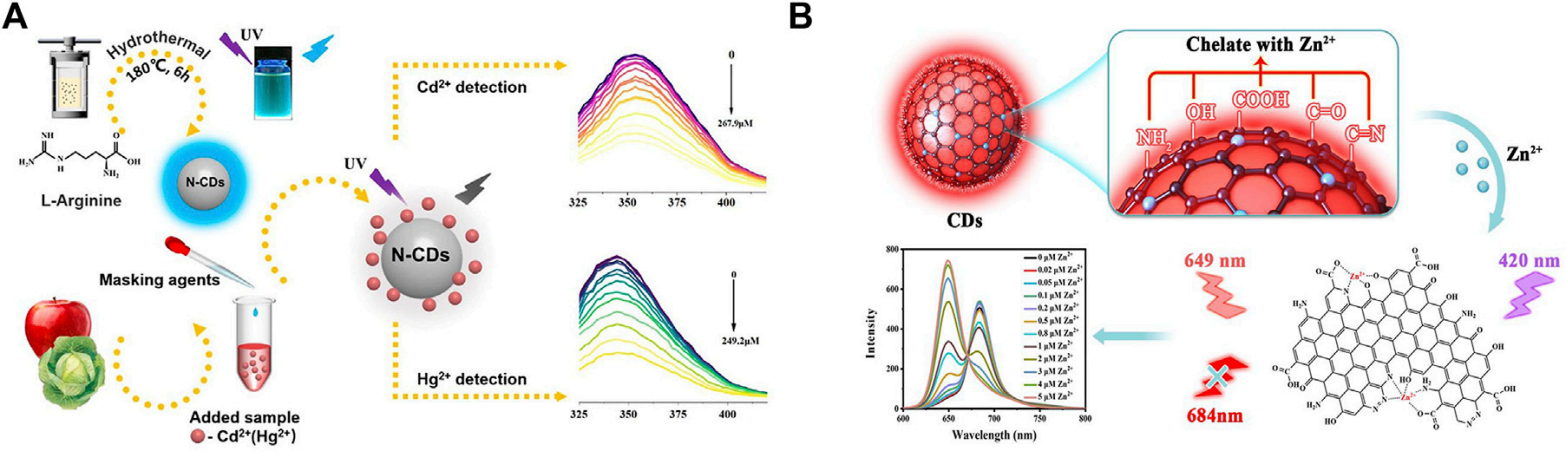
**(A)** The process of preparation of N-CDs, and schematic diagram of the detection of Cd(II) and Hg(II) in food samples by N-CDs, reproduced with permission from (Tan et al., 2022). **(B)** Mechanism illustration of the CDs for the detection of zinc ion, reproduced with permission from (Lu et al., 2023).

Carbon nanomaterials present unique properties due to quantum size effect, surface effect, and macroscopic quantum tunneling effect. Since the first fluorescent carbon nanoparticles were extracted from the cleavage of carbon nanotubes in 2004, carbon dot-like materials had attracted much attention (Xu et al., 2004). Heteroatom chemical doping could effectively modulate the physicochemical and photochemical properties of CDs. Zou et al. (2022) employed nitrogen-doped carbon quantum dots (N-CQDs) for highly selective detection of Hg(II). Based on the joint of dynamic and static quenching effect, the sensing system realized practical detection in sorghum and rice samples with the LOD of 0.8 μM. Zhang et al. (2023a) synthesized an N-doped blue-light CDs (N-BCDs) with high quantum yield. Cr(VI) quenched the fluorescence of N-BCDs through the internal filter effect. N-BCDs were used for the instantaneous detection of trace Cr(VI) in drinking water. As shown in Figure 2A, Tan (Tan et al., 2022) et al. prepared nitrogen-doped CDs (N-CDs) using a one-step hydrothermal method. The fluorescence of N-CDs could be quenched by Cd(II) and Hg(II) within 5 minutes, allowing the rapid and sensitive detection of Cd(II) and Hg(II) in fruits and vegetables, with the LOD of 0.20 and 0.188 μM, respectively.

The aforementioned N-CDs fluorescent sensors were based on turn-off methodologies. Wang et al. (2022) constructed a three-channel fluorescent array sensor based on N-doped CDs (NCDs) and gold nanoclusters for the rapid and accurate detection of Cd(II), Pb(II), and Hg(II) in traditional Chinese medicine, with the LOD of 0.15, 0.20, and 0.09 μM, respectively. In addition, owing to the complexity of food samples, the development of ratiometric sensors for the detection of heavy metal ions is necessary to attenuate or eliminate the effects of the external environment. As shown in Figure 2B, Lu et al. (2023) developed a ratiometric fluorescent sensor based on double-emitting fluorescent CDs, which could specifically recognize Zn(II). In the presence of Zn(II), the fluorescence intensity of CDs decreased at 684 nm and increased at 649 nm, enabling the accurate determination of Zn(II) in milk powder and zinc gluconate oral solution, with the LOD of 5 nM.

## 5 Conclusion and outlook

Due to the harmful effects of heavy metal ions even at low concentrations, ultra-sensitive and selective methods for the detection of heavy metal ions in food samples are highly desirable. In this manuscript, we review the recent progress and development trends in the nanomaterial-based sensors for detecting trace heavy metal ions in food samples. The design principles, sensing mechanisms, and applications of electrochemical, colorimetric, and fluorescent methods are presented in details. Even though these assays are promising in the detection of trace heavy metal ions, there is still space for improvement, especially in the practical applications. On the one hand, some of the sensors used to detect trace heavy metal ions were only applied to simple samples, such as drinking water rather than food samples. This limitation was due to the various interfering substances in complex food samples, which would bring some interferences in the sensing system. Therefore, apart from improving the sensitivity of the sensor, the anti-interference and selectivity of the sensor in complex matrices needs to be improved, so that these methods can be extensively applied to the detection of trace heavy metal ions in various foods. On the other hand, most of the currently developed sensors could only be applied to detect one or a few heavy metal ions. Sensors that can simultaneously detect various heavy metal ions are relatively lacking. We envision that with the development of different sensing arrays and the assistance of deep-learning algorithms, the sensitive and accurate detection of these heavy metal ions in high throughput will be achieved in the near future. The advancement of these sensing technologies will benefit the on-site precise and rapid screening of trace heavy metal ions for food safety concerns.
